# *TNFRSF1B *+676 T>G polymorphism predicts survival of non-Small cell lung cancer patients treated with chemoradiotherapy

**DOI:** 10.1186/1471-2407-11-447

**Published:** 2011-10-14

**Authors:** Xiaoxiang Guan, Zhongxin Liao, Hongxia Ma, Ji Qian, Zhensheng Liu, Xianglin Yuan, Daniel Gomez, Ritsuko Komaki, Li-E Wang, Qingyi Wei

**Affiliations:** 1 Department of Epidemiology, The University of Texas M. D. Anderson Cancer Center, 1515 Holcombe Blvd, Houston, Texas 77030, USA; 2 Department of Radiation Oncology, The University of Texas M. D. Anderson Cancer Center, 1515 Holcombe Blvd, Houston, Texas 77030, USA; 3 Department of Oncology, Tongji Hospital, Tongji Medical College, Huazhong University of Science and Technology, 13 Hangkong Lu, Wuhan 430030, China

**Keywords:** *TNF-α*, *TNFRSF1B*, polymorphism, non-small cell lung cancer, survival

## Abstract

**Background:**

The dysregulation of gene expression in the TNF-TNFR superfamily has been involved in various human cancers including non-small cell lung cancer (NSCLC). Furthermore, functional polymorphisms in *TNF-α *and *TNFRSF1B *genes that alter gene expression are likely to be associated with risk and clinical outcomes of cancers. However, few reported studies have investigated the association between potentially functional SNPs in both *TNF-α *and *TNFRSF1B *and prognosis of NSCLC patients treated with chemoradiotherapy.

**Methods:**

We genotyped five potentially functional polymorphisms of *TNF-α *and *TNFRSF1B *genes [*TNF-α *-308 G>A (rs1800629) and -1031 T>C (rs1799964); *TNFRSF1B *+676 T>G (rs1061622), -1709A>T(rs652625) and +1663A>G (rs1061624)] in 225 NSCLC patients treated with chemoradiotherapy or radiotherapy alone. Kaplan-Meier survival analysis, log-rank tests and Cox proportional hazard models were used to evaluate associations between these variants and NSCLC overall survival (OS).

**Results:**

We found that the *TNFRSF1B *+676 GG genotype was associated with a significantly better OS of NSCLC (GG *vs. *TT: adjusted HR = 0.38, 95% CI = 0.15-0.94; GG *vs. *GT/TT: adjusted HR = 0.35, 95% CI = 0.14-0.88). Further stepwise multivariate Cox regression analysis showed that the *TNFRSF1B *+676 GG was an independent prognosis predictor in this NSCLC cohort (GG *vs. *GT/TT: HR = 0.35, 95% CI = 0.14-0.85), in the presence of node status (N_2-3 _*vs. *N_0-1_: HR = 1.60, 95% CI = 1.09-2.35) and tumor stage (T_3-4 _*vs. *T_0-2_: HR = 1.48, 95% CI = 1.08-2.03).

**Conclusions:**

Although the exact biological function for this SNP remains to be explored, our findings suggest a possible role of *TNFRSF1B *+676 T>G (rs1061622) in the prognosis of NSCLC. Further large and functional studies are needed to confirm our findings.

## Background

Lung cancer is the most common tobacco-induced cancer and the leading cause of cancer-related deaths worldwide, with an estimated 1.61 million new cases and 1.38 million deaths in 2008 [1]. About 80% of primary lung cancer patients are non-small cell lung cancer (NSCLC), and one third of the patients were diagnosed at a locally advanced stage [2]. Despite significant advances in early detection and combination treatment including radiotherapy and chemotherapy in the last few decades, the prognosis of lung cancer remains poor, with a five-year overall survival rate of about 15% in the United States [3]. The tumor, lymph node, metastasis (TNM) staging system of lung cancer has been used as a guide for predicting prognosis [4]; however, dramatically different survival outcomes in NSCLC patients with the same pathological or clinical stage and the same treatments suggest that other factors may play an important role in the prognosis of NSCLC. Therefore, the discovery and application of novel prognostic biomarkers could help predict clinical outcomes and administer the optimal therapy in the management of NSCLC patients.

Tumor necrosis factor alpha (TNF-α) is a pro-inflammatory cytokine produced by activated macrophages and exerts its action through binding to its two cognate cell surface receptors, TNFRSF1A/TNFR1 (p55/60) and TNFRSF1B/TNFR2 (p75/80). It is well known that TNF and its superfamily members have both beneficial and harmful activities, playing a role as a "double-edged sword" [5]. Although TNF was discovered as a cytokine that could kill tumor cells, it is now clear that TNF can also contribute to tumorigenesis by mediating the proliferation, invasion and metastasis of tumor cells [5]. The dysregulation of gene expression in the TNF-TNFR superfamily has been reported to be involved in the development and prognosis of various human cancers including NSCLC [6-12]. For example, studies indicated that high serum concentrations of TNF were associated with a significantly longer survival in NSCLC patients after chemotherapy [12] and that TNFRSF1B had a significantly different expression profile in 5-FU-non-responding and responding liver cancer patients [11]. Additionally, recent reports found that TNF-α was involved in the pathogenesis of radiation-induced lung injury [13] and that inhibiting the TNF-α pathway was a novel radioprotection strategy [14]. These observations suggest that *TNF *and *TNFRSF1B *may play a role in patients' treatment response, toxicity, and survival. Thus, genetic variations in *TNF *and *TNFRSF1B *that alter gene expression and/or protein production may be potential candidates for prognosis predictors of NSCLC patients.

*TNF-α *and *TNFRSF1B *genes are highly polymorphic, and several functional single nucleotide polymorphisms (SNPs) in these two genes have been identified, which may contribute to differences in expression levels of the genes or protein products [15-20]. Of a particular significance are two *TNF-α *SNPs (SNP -308 G>A and -1031 T>C in the promoter region) and one *TNFRSF1B *SNP (+676 T>G in exon 6), which have been widely investigated for their associations with susceptibility to and progression and prognosis of various cancers [21-37]. However, to the best of our knowledge, no published study has investigated associations between potentially functional SNPs of these two genes and prognosis of NSCLC patients treated with chemoradiotherapy. Therefore, we performed a case-only study with 225 NSCLC patients treated with chemoradiotherapy or radiotherapy alone to investigate whether these three SNPs (SNP -308 G>A and -1031 T>C in *TNF-α*, and +676 T>G) as well as the other two potentially functional SNPs (-1709A>T and +1663A>G in *TNFRSF1B*) are associated with overall survival of NSCLC.

## Methods

### Study population

Epidemiological and clinical data were available from a larger dataset of 576 NSCLC patients who were treated with definitive radiation at The University of Texas M.D. Anderson Cancer Center (Houston, TX) between 1998 and 2006. The detailed information about these subjects has been described elsewhere [38]. After the exclusion of those patients who had surgical resection or had been treated elsewhere before coming to M. D. Anderson, a total of 225 NSCLC patients treated with chemoradiotherapy or radiotherapy alone were included in this analysis; The study was approved by the M. D. Anderson Cancer Center institutional review board in compliance with Health Insurance Portability and Accountability Act (HIPAA) regulations.

### SNP selection

We screened the NCBI dbSNP database (http://www.ncbi.nlm.nih.gov/, build 131) and Hapmap database (http://www.hapmap.org/, Rel 27) for common, potentially functional SNPs with a minor allele frequency (MAF) ≥ 0.05 in CEU populations, i.e., those SNPs located in the 5' near gene, 5'- and 3'-untranslated regions and coding region of *TNF-α *and *TNFRSF1B*. Meanwhile, linkage disequilibrium (LD) analysis and bioinformatics prediction by SNPinfo (http://snpinfo.niehs.nih.gov/) [39] were also performed to optimize SNP selection. Finally, a total of five SNPs (*TNF-α *-308 G>A, *TNF-α *-1031 T>C, and *TNFRSF1B *+676 T>G, *TNFRSF1B *-1709A>T and *TNFRSF1B*+1663A>G) were selected for genotyping. Among these, three SNPs are located in the promoter regions of the genes (*TNF-α *-308 G>A, *TNF-α *-1031 T>C, and *TNFRSF1B *-1709A>T), which are predicted to affect the binding of some transcription factors. One SNP (*TNFRSF1B *+676 T>G) causes a missense change and the other (*TNFRSF1B *+1663A>G) is located in the 3'-untranslated region, which may affect miRNA binding.

### Genotyping

Genomic DNA was isolated from the buffy coat fraction of each blood sample with a Blood Mini Kit (Qiagen, Valencia, CA) according to the manufacturer's instructions. Genotypes of the three selected SNPs (SNP -308 G>A and -1031 C>T in *TNF-α*, and -1709A>T in *TNFRSF1B *) were determined by the primer-introduced restriction analysis (PIRA)-PCR assay. The primers used for *TNF-α *-308 G >A, *TNF-α *-1031 T>C and *TNFRSF1B *-1709A>T were 5'-GCAATAGGTTTTGAGG*G*CCATG-3' (sense) and 5'-TTTGGAAAGTTGGGGACACACA-3'(anti-sense); 5'-GGGAAGCAAAGGAGAAGCTGAGAA*C*A-3' (sense) and 5'-GGGGGGTCCCCATACTCGACTT-3' (anti-sense); and 5'-CCACACACCTCAAGACCAATG*A*G-3' (sense) and 5'-CTTGAATTCGTTCCCAGGATGG-3' (anti-sense), respectively. The mismatched G, C and A were respectively introduced into the sense primers at 6 bp, 2 bp and 2 bp from these three polymorphic sites to create *Nco*I, *Nla*III and *Mly*I restriction sites. Additionally, *TNFRSF1B *+676T>G and +1663A>G were genotyped by the polymerase chain reaction-restriction fragment length polymorphism (PCR-RFLP) method with the primers 5'-CTCCTCCTCCAGCTGTAACG-3' (sense) and 5'-CCAACTGGAAGAGCGAAGTC-3' (anti-sense), and 5'-AGGCCCCCACCACTAGGACTCT-3' (sense) and 5'-GTTGTGGAAAGCCTCTGCTGC-3' (anti-sense), respectively. The following PCR conditions were performed: 5 min of initial denaturation at 95°C followed by 35 cycles of 30 s at 95°C; 45 s at 64°C (*TNF-α *-308 G >A) or 59°C (*TNF-α *-1031 C>T) or 64°C (*TNFRSF1B *+676 T>G) or 59°C (*TNFRSF1B *-1709A>T) or 62°C (*TNFRSF1B *+1663A>G), respectively; 45 s at 72°C; and a final 10-min step at 72°C for final extension.

**Figure 1:**
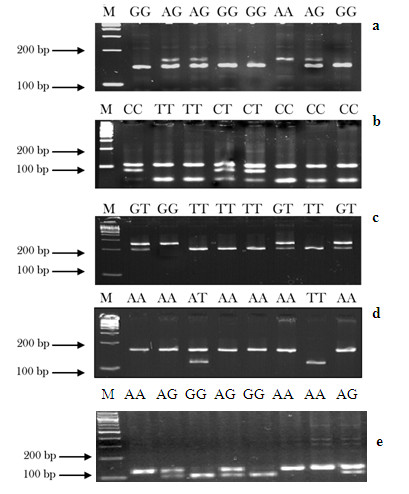
**PCR-based genotyping for *TNF*-*α *-308 G>A (A), *TNF-α *-1031C>T (B), *TNFRSF1B *+676 G>T (C), and *TNFRSF1B *-1709 A>T (D)**.

All the PCR products were digested with the restriction enzymes overnight (*Nco*I for *TNF-α *-308 G >A, *Nla*III for *TNF-α *-1031 C>T and *TNFRSF1B *+676 T>G, and *Mly*I for *TNFRSF1B *-1709A>T) and *Msp*A1I for *TNFRSF1B *(+1663A>G), and the products were separated in 3% MetaPhor agarose gel. After completion of gel electrophoresis, the *TNF-α *-308 GG genotype produced two bands (127- and 18-bp), AA produced a single band (145-bp), and AG displayed all three bands (145-, 127-, and 18-bp) (Figure 1A); the *TNF-α *-1031 CC genotype resulted in two bands of 108- and 86-bp, TT produced three bands of 108-, 62- and 24-bp, and CT produced four bands (108-, 86-, 62- and 24-bp) (Figure 1B); the *TNFRSF1B *+676 TT genotype produced two bands (200- and 48-bp), GG produced a single band (248-bp), and TG displayed all three bands (248-, 200- and 48-bp) (Figure 1C); *TNFRSF1B *-1709 TT genotype produced two bands of 136- and 24-bp, AA resulted in a single 160-bp band, and TA displayed all three bands (160-, 136- and 24-bp) (Figure 1D); and *TNFRSF1B *+1663AA resulted in a single 128-bp band, GG genotype produced another single band of 104-bp, and GA displayed all two bands (128-, and 104-bp) (Figure 1E)

Genotyping was performed without knowing the subjects' disease status. Two research assistants independently read the gel pictures and performed the repeated assays, if they did not reach a consensus on the tested genotype. About 10% of the samples were randomly selected to perform the repeated assays, and the results were 100% concordant.

### Statistical analysis

The two-sided χ^2 ^tests were performed to determine any statistically significant differences in the distributions of the *TNF-α *and *TNFRSF1B *genotypes by demographic variables and clinical features. Median survival time (MST) was calculated, and mean survival time was presented when the MST could not be calculated. Kaplan-Meier estimates were used to estimate overall survival (OS) among four genotype groups, and the log-rank test was used to test for equality of the survival distributions. Univariate analysis and multivariate Cox proportional hazards models were conducted to estimate the effect of each genotype on survival with or without the presence of known prognostic factors. Cox stepwise hazards regression model was also conducted to determine independently predictors of NSCLC prognosis, with a significance level of 0.050 for entering and 0.051 for removing the respective explanatory variables. Haplotype frequencies and individual haplotypes based on the observed genotypes were generated by using SAS PROC HAPLOTYPE. Analyses were performed by using SAS statistical package version 9.1 (SAS Institute Inc., Cary, NC).

## Results

### Patient characteristics and clinical features

As shown in Table 1, 225 patients were included in the final analyses, of which 155 deaths were observed during the follow-up period, and the overall MST was 23 months. Among the cases, 79 (35.1%) were adenocarcinoma, 71 (31.6) squamous cell carcinoma, and 75 (33.3%) other type of NSCLC. According to the AJCC 6^th ^edition stage grouping criteria [40], 33 cases (14.7%) were stage I-II, and 192 (85.3%) were stage III-IV. In this study, all cases received radiotherapy with a median radiation dose of 63.0 (range 50.4-84.0) Gy at 1.8 to 2 Gy per fraction once a day. Platinum and taxane-based chemotherapy was also given to 163 (72.4%) patients. None of the patients received surgery. Log-rank test showed that, in this study population, there were no significant differences in NSCLC-specific survival by age (log-rank *P *= 0.117), ethnicity (*P *= 0.827), smoking status (*P *= 0.457), histology (*P *= 0.092), Karnofsky's performance scores (KPS, *P *= 0.405), chemotherapy status (*P *= 0.407) and radiotherapy dose (*P *= 0.706). However, sex, tumor stage, and node status were significantly associated with MST of NSCLC (log-rank *P *= 0.036, 0.010 and 0.023, respectively). Because these variables may be confounding factors for the effect of the genotypes on OS, they were further adjusted in the multivariable analysis.

**Table 1:** Characteristics of patients (N = 225) and overall survival (OS)

Variables	Patients No. (%)	Deaths No. (%)	MST (Months)	Log-rank *P*^a^
Age				
≤62 years	111 (49.3)	72 (46.5)	25.6	0.117
>62 years	114 (50.7)	83 (53.5)	21.1	
Gender				
Female	102 (45.3)	65 (41.9)	26.7	0.036
Male	123 (54.5)	90 (58.1)	22.3	
Ethnicity				
White	162 (72.0)	117 (75.5)	23.5	0.827
Non-White	63 (28.0)	38 (24.5)	22.3	
Smoke^b^				
Ever	205 (91.5)	143 (92.9)	23.4	0.457
Never	19 (8.5)	11 (7.1)	21.1	
Histology				
Adenocarcinoma	79 (35.1)	48 (31.0)	27.3	0.092
Squamous cell	71 (31.6)	51 (32.9)	23.2	
Others ^b^	75 (33.3)	56 (36.1)	19.0	
KPS^c^				
90-100	51 (25.4)	34 (22.7)	25.1	0.405
80	118 (58.7)	90 (60.0)	22.1	
<80	32 (15.9)	26 (17.3)	21.1	
Tumor stage ^c^				
T_0-2_	127 (57.2)	81 (52.3)	26.9	0.010
T_3-4_	95 (42.8)	74 (47.7)	19.2	
Node status ^c^				
N_0-1_	59 (26.7)	35 (22.6)	29.0	0.023
N_2-3_	162 (73.3)	120 (77.4)	20.7	
Chemotherapy				
Yes	163 (72.4)	113 (72.9)	26.9	0.407
No	62 (27.6)	42 (27.1)	22.1	
Radiotherapy dose^d^				
≤63.0 Gy	115 (51.1)	77 (49.7)	22.7	0.706
>63.0 Gy	110 (48.9)	78 (50.3)	25.1	

MST: median survival time; KPS: Karnofsky's performance scores

^a ^Log-rank test.

^b ^Others include large cell, undifferentiated and mixed-cell carcinomas.

^c ^Some missing data.

^d^The median radiotherapy dose is 63 Gy.

### *TNF-α *and *TNFRSF1B *genotypes and NSCLC survival

**Figure 2:**
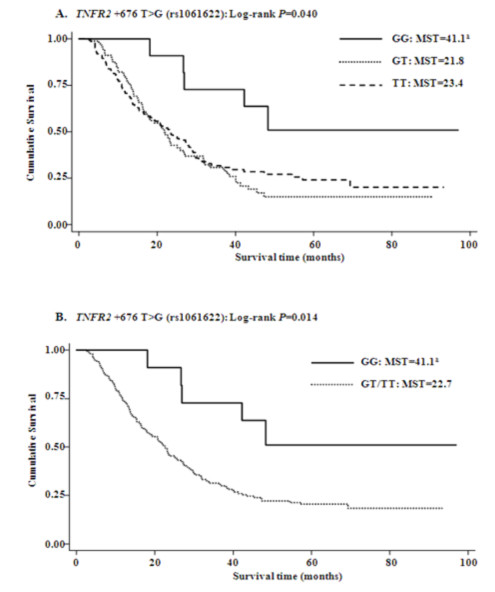
**Overall survival curves by genotypes of *TNFRSF1B *+676 T>G (rs1061622) in additive model (A) and recessive model (B)**. The *P *values were obtained from the unadjusted log-rank test. ^a ^Mean survival time was provided when MST could not be calculated.

The genotype distributions of the five SNPs in *TNF-α *and *TNFRSF1B *and their associations with OS are summarized in Table 2. In all patients, only genotypes of *TNFRSF1B *+676 G>T (rs1061622) were statistically significantly associated with OS (log-rank *P *= 0.040 in an additive model, Figure 2A; log-rank *P *= 0.014 in a recessive model, Figure 2B). After adjustment for age, sex, ethnicity, smoking status, tumor histology, Karnofsky's performance scores, tumor stage, application of chemotherapy and radiotherapy dose, the *TNFRSF1B *+676 GG variant homozygous genotype remained to be associated with a significantly decreased risk of death from NSCLC (adjusted HR = 0.38, 95% CI = 0.15-0.94 for GG *vs. *TT; adjusted HR = 0.35, 95% CI = 0.14-0.88 for GG *vs. *GT/TT), but this association was not observed for the other four SNPs investigated in this study.

**Table 2:** Associations between *TNF-α *and *TNFRSF1B *genotypes and overall survival of NSCLC patients

Genotypes	No. of patients	No. of Deaths	MST (months)	Crude HR (95% CI)	Adjusted HR (95% CI)^a^	Adjusted *P*^a^
*TNF-α *-308 G>A (rs1800629)						
GG	163	112 (68.7)	23.5	1.00	1.00	
AG	56	39 (69.6)	23.2	1.04 (0.72-1.50)	0.95 (0.65-1.40)	0.802
AA	6	4 (66.7)	18.1	1.18 (0.44-3.21)	1.10 (0.39-3.11)	0.859
AG+AA	61	43 (70.5)	23.1	1.05 (0.74-1.50)	0.96 (0.67-1.40)	0.849
AG+GG	219	151 (68.9)	23.4	1.00	1.00	
AA	6	4 (66.7)	18.1	1.17(0.43-3.16)	1.11 (0.40-3.13)	0.840
*TNF-α *-1031T>C (rs1799964)						
TT	137	95 (69.3)	23.4	1.00	1.00	
CT	77	50 (64.9)	22.1	0.96 (0.68-1.35)	0.89 (0.62-1.28)	0.542
CC	11	10 (90.9)	23.1	1.28 (0.67-2.46)	1.41 (0.70-2.87)	0.341
CT+CC	88	60 (68.2)	23.1	1.00 (0.72-1.38)	0.95 (0.68-1.34)	0.785
CT+TT	214	145 (67.8)	23.4	1.00	1.00	
CC	11	10 (90.9)	23.1	1.30(0.68-2.47)	1.46 (0.73-2.94)	0.289
*TNFRSF1B *+676 T>G (rs1061622)						
TT	124	85 (68.5)	23.4	1.00	1.00	
GT	90	65 (72.2)	21.8	1.10 (0.80-1.52)	1.19 (0.84-1.69)	0.323
GG	11	5 (45.5)	41.1^b^	0.36 (0.14-0.88)	0.38 (0.15-0.94)	0.037
GT+GG	101	70 (69.3)	23.2	0.96 (0.70-1.31)	1.01 (0.72-1.42)	0.937
GT+TT	214	150 (70.1)	22.7	1.00	1.00	
GG	11	5 (45.5)	41.1^b^	0.34 (0.14-0.84)	0.35 (0.14-0.88)	0.026
*TNFRSF1B *-1709A>T (rs652625)						
AA	207	144 (69.6)	23.2	1.00	1.00	
AT	15	9 (60.0)	27.8	0.86 (0.44-1.69)	0.85 (0.42-1.71)	0.649
TT	3	2 (66.7)	15.1	0.94 (0.23-3.82)	1.02 (0.23-4.47)	0.976
AT+TT	18	11 (61.1)	26.7	0.88 (0.47-1.62)	0.87 (0.46-1.67)	0.685
AT+AA	222	153 (68.9)	23.4	1.00	1.00	
TT	3	2 (66.7)	15.1	0.95 (0.24-3.85)	1.05 (0.24-4.58)	0.946
*TNFRSF1B *+1663A>G(rs1061624)						
AA	67	46 (68.7)	23.4	1.00	1.00	
AG	89	63 (70.8)	21.1	1.06 (0.73-1.53)	0.98 (0.68-1.43)	0.921
GG	62	41 (66.1)	28.1	0.84 (0.56-1.26)	0.79 (0.52-1.20)	0.269
AG+GG	151	104 (68.9)	24.0	1.03(0.73-1.46)	0.96 (0.67-1.37)	0.824
AG+AA	156	109 (69.9)	22.7	1.00	1.00	
GG	62	41 (66.1)	28.1	0.84 (0.59-1.20)	0.81 (0.56-1.18)	0.275

MST: median survival time; HR: hazard ratio

^a^Adjusted for age, gender, ethnicity, smoking status, tumor histology, KPS, tumor stage, node status, application of chemotherapy and radiotherapy dose.

^b^Mean survival time was provided when MST could not be calculated.

In order to confirm an independent role of SNPs in NSCLC survival, we further performed a multivariate stepwise analysis with selected demographic characteristics and clinical features in different genetic models of *TNFRSF1B *+676 T>G (rs1061622) on NSCLC survival. Three variables (tumor stage, node status and rs1061622 GG *vs. *GT/TT) were identified in the regression model with a significance level of 0.050 for entering and 0.051 for removing a variable (Table 3), suggesting the independent effect of these three factors on death risk of NSCLC. When age and sex were forced into the final model, the rs1061622 GG genotype remained a significantly favorable predictor for NSCLC survival (GG *vs. *GT/TT: HR = 0.39, 95% CI = 0.16-0.95) (Table 3).

**Table 3:** Results of stepwise Cox regression analysis on NSCLC

Variables	*β*	SE	HR	95% CI	*P*
*Stepwise Regression Analysis*					
Node status (N_2-3 _*vs. *N_0-1_)	0.472	0.195	1.60	1.09-2.35	0.015
Tumor stage (T_3-4 _*vs. *T_0-2_)	0.392	0.162	1.48	1.08-2.03	0.016
*TNFRSF1B *+676 T>G (GG *vs *GT/TT)	-1.061	0.457	0.35	0.14-0.85	0.020
*Final Regression Model*					
Age (>62 years *vs. *≤62 years)	0.288	0.165	1.33	0.97-1.84	0.081
Sex (Male *v.s *Female)	0.252	0.165	1.29	0.93-1.78	0.126
Node status (N_2-3 _*vs. *N_0-1_)	0.511	0.196	1.67	1.14-2.45	0.009
Tumor stage (T_3-4 _*vs. *T_0-2_)	0.392	0.163	1.48	1.08-2.04	0.016
*TNFRSF1B *+676 T>G (GG *vs *GT/TT)	-0.954	0.459	0.39	0.16-0.95	0.038

SE: standard error; HR: hazard ratio

### Stratification analysis and haplotype analysis

**Figure 3:**
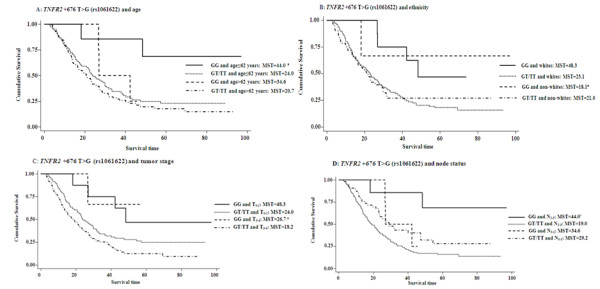
**Overall survival curves by genotypes of *TNFRSF1B *+676 T>G (rs1061622) and selected factors including age (A), ethnicity (B), tumor stage (C) and node status(D)**. The *P *values were obtained from the unadjusted log-rank test.^a ^Mean survival time was provided when MST could not be calculated.

As shown in Figure 3A-E, we further evaluated cumulative survival in subgroups by *TNFRSF1B *+676 T>G (rs1061622) genotypes and selected factors including age, ethnicity, tumor stage and node status. The longest survival was observed in subgroups with both rs1061622 GG and age≤62 years (MST = 44.0 months), or white ethnicity (MST = 48.3 months), or T_0-2 _tumors (MST = 48.3 months), or N_2-3 _tumors (MST = 44.0 months), compared with the poorest survival in subgroups with both rs1061622 GT/TT and age>62 years (MST = 20.7 months), or non-white (MST = 21.0 months), or T_3-4 _tumors (MST = 18.2 months), or N_0-1 _tumors (MST = 29.2 months); however, small sample size in these subgroups might have led to a suboptimal statistical power to detect a statistical difference in the survival time. We also conducted the stratification analysis by histology and different treatments, but the numbers of patients in subgroups were too small, and no significant results were found in every stratum (data not shown).

In addition, we also evaluated the combined effect of these polymorphisms on NSCLC survival by using the haplotype analysis. The results showed that *TNF-α *-308G/-1031T (GT) and *TNFRSF1B *+676T/+1663A/-1709A (TAA) were the most common haplotypes in the patients with the frequencies of 63.3% and 40.2%, respectively. However, we did not found significant associations between other haplotypes and OS of NSCLC patients, compared to the most common haplotypes (data not shown), which is likely due to limited study power.

## Discussion

In the present study, we examined the effect of five selected polymorphisms in *TNF-α *and *TNFRSF1B *on survival of NSCLC patients treated with chemoradiotherapy or radiotherapy alone. We found that the *TNFRSF1B *+676 GG (rs1061622) variant homozygous genotype was associated with a significantly improved survival of NSCLC in this non-Hispanic patient population. Such an effect was not observed for other SPNs under investigation.

Tumor necrosis factor-alpha (TNF-α) executes multiple functions in immunity, inflammation, differentiation, control of cell proliferation, and apoptosis through distinct receptors known as TNF receptor type I (TNFR1) and type II (TNFR2) [5]. Expression levels of TNF-α and its receptors have been linked to development and treatment outcomes of solid tumors including NSCLC [6,7,10]. The expression of *TNF-α *is mostly regulated at the transcriptional level, and polymorphisms within the *TNF-α *promoter have been related to TNF-α levels [15,23]. For example, *TNF-α-308 *G/A in the promoter of *TNF-α *has been reported to be associated with higher expression levels of *TNF-α *[41] and thus susceptibility to numerous cancers, including cancers of the stomach [25], breasts [34], oral cavity [31,32], bladder [35] and lung [25]. Additionally, several studies also found that *TNF-α-308 *G/A and another SNP in the promoter of *TNF-α *(-1031C>T, rs1799964) were significantly associated with prognosis of bladder cancer and breast cancer [28,29,36], clinical characteristics of colorectal cancer, and severity of lung cancer [25]. However, no significant associations between these two SNPs and NSCLC survival were found in our study, which was consistent with recent data that suggested the lack of associations between *TNF-α-308 *G/A or *TNF-α-1031 *T>C and the prognosis of several other cancers, including gastric cancer [42], colorectal cancer [43], Hodgkin's lymphoma [44], and breast cancer [45]. Such a discrepancy of reported studies might be explained by different tumor sites and tumor progression features, ethnic difference with diverse genetic background, different therapeutic strategies, and either false negative or false positive results because of the small sample sizes in previously published studies.

Interestingly, logistic and stepwise regression analysis in our study indicated that *TNFRSF1B *rs1061622 GG might be an independent predictor for survival of NSCLC patients treated with chemoradiotherapy or radiotherapy alone. The TNFRSF1B is the principal TNF receptor found on circulating T lymphocytes, which functions both as a TNF antagonist by neutralizing it and as an agonist by facilitating interaction between TNF and TNFR1 at the cell surface [46]. rs1061622 is located in the exon 6 of *TNFRSF1B *(M196R) that causes a functional amino acid change from methionine (M) to arginine (R) [20], which is postulated to affect the proteolytic cleavage of the membrane bound TNFRSF1B to a soluble form as well as TNF binding and/or TNF induced apoptosis by impaired NF-κB signaling [47,48].

However, the results from published studies on associations between rs1061622 and prognosis of cancers were controversial rather than conclusive. For example, a Tunisian study showed significant associations of *TNFRSF1B *+676 T>G (rs1061622) with susceptibility and survival of breast cancer patients [26], but another Japanese study suggested no association between this SNP and prognosis of esophageal squamous cell carcinoma [49]. Up to now, no published study investigated the association between *TNFRSF1B *+676 T>G (rs1061622) and lung cancer survival, and the exact biological mechanism underlying this association in our study remains to be investigated. It is possible that this SNP affects the function of *TNFRSF1B *and enhances radiotherapy or chemotherapy efficacy through inhibiting tumor therapy resistance. Another possibility is that this SNP may change the expression level of *TNFRSF1B *and influence the character of immunological response to infection in lung cancer patients, finally resulting in the difference of survival in NSCLC [50]. Furthermore, we also found that cumulative survival was different in subgroups by *TNFRSF1B *+676 T>G (rs1061622) genotypes and selected factors including age, ethnicity, tumor stage and node status. Although our sample size was relatively small in these subgroups, it provided a clue that effect of this SNP on prognosis of NSCLC may be dependent on patients' characteristics and clinical features as well. However, the exact functional relevance of this SNP in the prognosis of NSCLC patients treated with chemoradiotherapy remains to be explored.

Although our study reported some new findings about polymorphisms of *TNF *and *TNFRSF1B *genes in NSCLC patients, several potential limitations should be taken into consideration. Firstly, our sample size is relatively small with a limited statistical power to evaluate interactions between the studied polymorphisms and clinical factors including tumor stage and node status. For the same reason, the results from our subgroups by *TNFRSF1B *+676 T>G (rs1061622) genotypes and selected factors need validation by larger studies. Secondly, only five potentially functional SNPs in *TNF *and *TNFRSF1B *were included in our study, which is far from comprehensive. Indeed, both genes are highly polymorphic, and it is possible that some important SNPs may have been missed or the observed association may have been due to other polymorphisms in LD with the studied ones. Again, much larger studies would have the statistical power to confirm the associations of more SNPs with OS in such a patient population.

## Conclusion

In summary, in this cohort of NSCLC patients, we identified a possible role of *TNFRSF1B *+676 T>G in the prognosis of NSCLC. However, replication studies with diverse ethnic groups, larger sample sizes and functional characterizations are warranted. Currently, we have ongoing projects that help continue recruiting similar patients for additional analysis in the future.

## Abbreviations

**TNF-α**: Tumor necrosis factor-alpha; **TNFR2**: tumor necrosis factor receptor super family 2; **PCR-RFLP**: polymerase chain reaction-restriction fragment length polymorphism; **HR**: hazard ratio; **CI**: confidence interval; **SNP**: single nucleotide polymorphism; **NSCLC**: non-small cell lung cancer; **OS**: overall survival.

## Competing interests

The authors declare that they have no competing interests.

## Authors' contributions

XXG, QYW and ZXL designed the study and prepared the manuscript. XXG performed the experiments with the assistance from ZSL and JQ. XXG and HXM conducted the analysis with assistance from LEW. XLY, DG and RK helped to collect and organize the clinical data. HXM, QYW and LEW helped to finalize the manuscript. All authors read and approved the final manuscript.

## Pre-publication history

The pre-publication history for this paper can be accessed here:

http://www.biomedcentral.com/1471-2407/11/447/prepub
